# CircNUP98 Suppresses the Maturation of miR-519a-3p in Glioblastoma

**DOI:** 10.3389/fneur.2021.679745

**Published:** 2021-11-18

**Authors:** Jun Lu, Gaojie Lou, Lin Jiang, Xiaoxing Liu, Jianxin Jiang, Xiaolin Wang

**Affiliations:** Department of Neurosurgery, Taizhou People's Hospital, Taizhou, China

**Keywords:** glioblastoma, circNUP98, miR-519a, proliferation, maturation

## Abstract

Circular RNA (circNUP98) has been reported to promote renal cancer; however, its role in other cancers is unknown. The function of circNUP98 in glioblastoma (GB) cancer was explored in this study. A total of 58 GB tissue samples were collected to study the expression of circNUP98 and miR-519a-3p [both the mature and pre-mature microRNA (miRNA)] by quantitative real-time PCR (RT-qPCR) and heatmap analysis. The subcellular location that expresses circNUP98 was analyzed by nuclear fractionation assay. RNA pull-down assay was performed to evaluate the interaction between circNUP98 and pre-mature miR-519a-3p. Overexpression assays were performed to investigate the role of circNUP98 in the regulation of both the mature and pre-mature miR-519a-3p. The role of circNUP98 and miR-519a-3p in GB cell proliferation was explored by 5-bromo-2-deoxyuridine (BrdU) assay and was assessed in mouse xenograft model. Heatmap analysis showed that circNUP98 and pre-mature miR-519a-3p were upregulated in GB, while mature miR-519a-3p was downregulated in GB. Across the cancer tissues, circNUP98 was inversely correlated with mature miR-519a-3p, but positively correlated with pre-mature miR-519a-3p. In GB cells, circNUP98 was localized to both the nucleus and cytoplasm and it interacted with pre-mature miR-519a-3p. In GB cells, circNUP98 increased the expression levels of pre-mature miR-519a-3p and decreased the expression levels of mature miR-519a-3p. BrdU and cholecystokinin octapeptide (CCK-8) assays illustrated that overexpression of circNUP98 reduced the inhibitory effects of miR-519a-3p on cell proliferation. CircNUP98 contributed to larger tumors, which resulted in significantly reduced mice survival. CircNUP98 suppresses the maturation of miR-519a-3p to promote GB cell proliferation.

## Introduction

Glioblastoma (GB), also commonly known as GB multiforme (GBM), is a highly aggressive malignancy that originates from either spinal cord or the brain (1). Although people in any age groups can develop GBM, this malignancy mainly affects patients older than 65 years old (2, 3). GBM may cause seizures, vomiting, nausea, and worsening headaches (4). Surgical resection followed by radiation therapy or oral chemotherapy is usually applied in the treatment of GBM (5). However, even with timely and effective treatment, only 25 and 5% of patients can survive for more than 1 and 5 years, respectively, and the average survival time is only 12–18 months (6, 7). Even worse, with the growth of aging population, incidence of GBM is increasing, leading to a serious threat to public health (8). Therefore, improving of GBM treatment is of great importance.

Chemotherapy and radiation therapy cause significant side effects and are not suitable for many cases of patients with GBM (9). With the advantages of specific targeting of cancer cells and less side effects, targeted therapies are emerging novel anticancer approaches that regulate gene expression network involved in human diseases, such as cancers, to achieve therapeutic approaches (10, 11). For example, regulating the expression of the epidermal growth factor receptor (EGFR) is a promising target for the treatment of GBM (12). Although advances have been made in GBM-targeted therapy, targets, safer, and more effective targets are still needed to improve GBM treatment. Circular RNAs (circRNAs) are a class of self-ligated RNAs with no or limited protein-coding capacity. CircRNAs have been found to be involved in many biological processes. For example, circRNA-vgll3 can promote osteogenic differentiation of adipose-derived mesenchymal stem cells (13) and circRNA Cerebellar-degeneration-related protein 1 antisense RNA (CDR1as) can regulate the proliferation of human periodontal ligament stem cells (14) In addition, cicRNAs have been found to be involved in the development of cancer by regulating the expression of targeted genes (15, 16). Therefore, certain circRNAs could be targeted to treat GBM. CircNUP98 is a novel identified circRNA, which is derived from the *NUP98* gene. A recent study has found that circNUP98 was significantly upregulated in renal cell carcinoma (RCC) and knockdown of circNUP98 inhibited RCC cell proliferation, migration, and invasion (17). However, it has a role in GB is unknown. MiR-519a-3p has a critical role in many cancers. For example, the downregulation of miR-519a-3p can enhance tumor progression in gastric cancer (18) and it can promote tumor growth in hepatocellular carcinoma (19). Previous studies have found that miR-519a plays an anticancer role in GB by regulating GBM cell proliferation, migration, and invasion (20). Our preliminary analysis showed that pre-mature miR-519a-3p was predicted to be a target of circNUP98. Therefore, in this study, we aimed to investigate the role of circNUP98 in GB and whether it functions by regulating miR-519a-3p.

## Materials and Methods

### Tissue Samples

This study enrolled 58 patients with GBM (38 males and 20 females, 69.1 ± 6.6 years old) who were admitted at the Taizhou People's Hospital (ethics approval was obtained from the Ethics Committee of this hospital) between January 2018 and January 2020. GBM and paired non-cancer tissues (3 cm around tumors) were collected from each of the patient. All the patients were willing to donate tissue samples and signed the informed consent. All the tumors were located to the supratentorial region and tissue samples were either collected during surgical resection or through biopsies. Patients who had other clinical disorders or initiated therapies were excluded.

### Glioblastoma Multiforme Cells and Electric Transfection

Two GBM cell lines U87 and U-251 ATCC (Manassas, USA) were used for *in-vitro* functional assays and gene expression analysis. These two cell lines were cultured in Eagle's Minimum Essential Medium (ATCC, USA) containing 1 mM sodium pyruvate and 10% fetal bovine serum in a humidified atmosphere with 5% CO_2_ at 37°C. For cell transfection, pcDNA3.1-circNUP98 vector or miR-519a-3p mimic Invitrogen (Carlsbad, CA, USA) was transfected into U87 and U-251 cells by using the Gene Pulser Xcell Electroporation System Bio-rad (Hercules, CA, USA). 10 μg vector or 45 nM mimic was transfected into 1 × 10^6^ U87 and U-251 cells. Empty vector or negative control (NC) miRNA was transfected to serve as the NC groups. Subsequent experiments were performed at 48 h after transfection. Short hairpin RNAs (shRNAs) targeting circNUP98 were ordered from the GenePharma (Shanghai, China). Cloning, lentivirus production, and infection were performed following the instructions of the manufacturer.

### Ribonucleic Acid Samples

Ribonucleic acid isolation was performed by using Ribozol VWR Life Science (Solon, Ohio, USA). Genomic DNA removal was performed with DNase I (Invitrogen, USA). RNA separations were performed by using 5% urea-polyacrylamide gel electrophoresis (PAGE) gel for integrity analysis.

### RT-qPCR

The complementary DNA (cDNA) was synthesized by using the M-MLV Reverse Transcriptase Kit (ELK Biotechnology, Wuhan, China) with the following conditions: 42°C for 60 min, 85°C for 5 min, and hold at 4°C. Following the preparation of cDNA samples, qPCRs were performed to determine the expression levels of circNUP98, pre-mature miR-519a-3p, and mature miR-519a-3p. Glyceraldehyde-3-phosphate dehydrogenase (GAPDH) and U6 were selected for internal controls. PCR thermal cycling were as follows: 95°C for 1 min, followed by 40 cycles of 95°C for 15 s, 58°C for 20 s, and a final extension of 72°C for 45 s. The relative expression levels were calculated by using the 2^−Δ*ΔCT*^ method (21) and the samples were run in triplicate. The primer sequences are listed in Table 1.

**Table 1:** Primer sequences for RT-qPCR.

**Gene Name**	**Forward primer (5^′^-3^′^)**	**Reverse primer (5^′^-3^′^)**
CircNUP98	AGCACAGGGACCAGTCTTTTC	AGGCTTCCAGTATTGTTGCTG
Signal transducer and activator of transcription3 (STAT3)	ATCACGCCTTCTACA GACTGC	CATCCTGGAGATTCTCTACCACT
GAPDH	CCACTCCTCCACCTTTGAC	ACCCTGTTGCTGTAGCCA
Pre-mature miR-519a-3p	CTCAGGCTGTGACACTCTAG	AATCTCACAATGACAAACTC
Mature miR-519a-3p	AAAGTGCATCCTTTTAGAGTGT	GTGCAGGGTCCGAGGTATT
U6	GCGCGTCGTGAAGCGTTC	GTGCAGGGTCCGAGGT

### Ribonucleic Acid Pull-Down Assay

Biotinylated RNAs, including pre-mature miR-519a-3p wild-type (WT) (bio-pre-miR-519a-3p-WT: 5′- CUGUGACACUCUAGAGGGAAGCGC-3′), mature miR-519a-3p wild type (bio-pre-miR-519a-3p-Mut: 5′-CUGUGGGGCUCUACAAGAAAGCGC-3′), and NC miRNA (bio-NC: 5′-CGAUUGGCUCGAAGCGCGUCAAGA-3′), were purchased from the Invitrogen. For cell transfection, 1 × 10^6^ U87 and U-251 cells were transfected with 45 nM bio-pre-miR-519a-3p-WT, bio-pre-miR-519a-3p-Mut, or bio-NC. Cells were cultivated for 48 h after transfection followed by the preparation of cell lysate samples. The labeled RNAs were then pulled down by using streptavidin agarose magnetic beads Life Technologies (Gaithersburg, MD, USA). After that, total RNAs were isolated and reverse transcription was performed. Finally, RT-qPCR was performed with GAPDH as the internal control to determine the expression levels of circNUP98.

### Dual-Luciferase Assay

To detect the interaction between circNUP98 and miR-519a-3p, dual-luciferase assay was performed. The sequences of WT or mutated (MUT) circNUP98 were cloned into XbaI-FseI sites of pGL3 promoter. Then, the luciferase reporter plasmids were cotransfected with miR-519a-3p mimics into U87 and U-251 cells by using Lipofectamine 2000. After transfection for 48 h, cells were lysed, and the relative luciferase activities were detected by using the Dual-Luciferase Reporter Assay System Promega (Madison,WA, USA).

### Nuclear Fractionation Assay

Nucleus and cytoplam samples from U87 and U-251 cells were prepared by using the Nuclear/Cytosol Fractionation Kit (BioVision (Mountain View, CA, USA); #K266). Both the samples were subjected to RNA isolation followed by semi-quantitative PCRs to determine the expression of circNUP98. Data visualization was performed by separating PCR products using 1% agarose gels followed by image capturing under UV lights.

### BrdU Assay

BrdU incorporation was used to reflect cell proliferation. Following transfection, cells were cultivated in a 96-well plate with 5,000 cells per well and cultured at 37°C in 5% carbon dioxide (CO_2_). Cells were cultured for 48 h followed by incubation with 10 μM BrdU BD Pharmingen (San Diego, CA, USA) for 3 h. Following cell fixation, cells were incubated with peroxidase-coupled anti-BrdU antibody [ab6326; 1:200; Sigma-Aldrich (St. Louis, MO, USA)] at room temperature for 1 h. After that, cells were further incubated with 5 μM peroxidase substrate (tetramethylbenzidine) for another 30 min. Finally, optical density (OD) values at 450 nM were determined to reflect cell proliferation after the deduction of background values, which were the OD values of wells without BrdU treatment but with BrdU antibody incubation.

### *In-vivo* Intracranial Xenograft Tumor Models

All the animal experiments were approved by the Animal Ethics Committee of Taizhou People's Hospital and were performed according to the Guide for the Care and Use of Laboratory Animals. A 6-week-old female nude mice were randomly divided into two groups (*n* = 6/group). U87 cells with the overexpression of circNUP98 or control U87 cells were diluted to the density of 1.5 × 10^5^ cells in 2 μl phosphate buffered saline (PBS) and then injected into the brain. Mice were monitored until the following events were observed: severe central neural system invasion symptoms such as paralysis, arched back, or dead.

### Statistical Analyses

All the experiments were repeated three times and the data were shown as mean ± SD. Average values of gene expression levels in three technical replicates were inputted into Heml 1.0 Software to generate heatmaps, which reflect differential gene expression levels. Two independent groups were compared by the unpaired *t*-test. The ANOVA Tukey's test was used to compare the multiple independent groups. Differences were statistically significant when *p* < 0.05.

## Results

### Analysis of the Expression of CircNUP98 and miR-519a-3p in Paired Tissues

**Figure 1:**
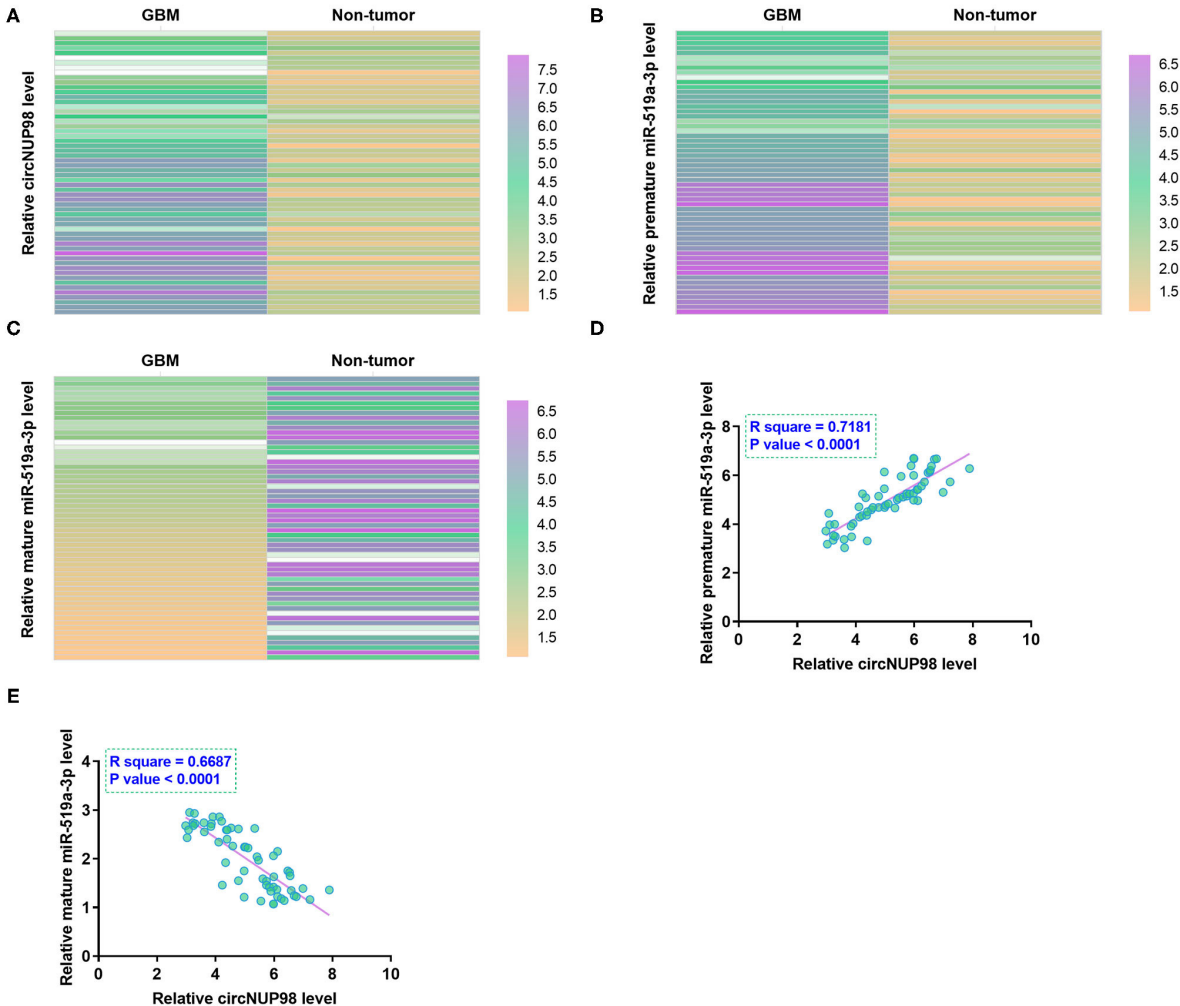
Analysis of the expression of circNUP98 and miR-519a-3p in paired tissues. RT-qPCR assays were performed to determine the differential expression of circNUP98 **(A)**, pre-mature miR-519a-3p **(B)**, and mature miR-519a-3p **(C)** in paired tissues from patients with glioblastoma multiforme (GBM) (*n* = 58). Differential gene expression was reflected by heatmaps, which were plotted by using the Heml 1.0 Software (Huazhong University of Science and Technology, Hubei, Wuhan, China). Correlation analysis was performed by using the Pearson's correlation coefficient to analyze the correlations between circNUP98 and mature miR-519a-3p **(D)** or pre-mature miR-519a-3p **(E)** across GBM tissue samples.

RT-qPCRs were performed to determine the differential expression of circNUP98 and miR-519a-3p (both the mature and pre-mature) in paired tissues from patients with GBM (*n* = 58). Heatmap analysis showed that circNUP98 (Figure 1A) and pre-mature miR-519a-3p (Figure 1B) were upregulated in GB tissues, while mature miR-519a-3p (Figure 1C) was downregulated in GB tissues. Therefore, upregulation of circNUP98 and the suppressed maturation of miR-519a-3p may be related to GBM. The Pearson's correlation coefficient showed that the expression of circNUP98 was inversely correlated with the expression of mature miR-519a-3p (Figure 1D), but positively correlated with the expression of pre-mature miR-519a-3p (Figure 1E) across GBM tissue samples.

### Subcellular Location of CircNUP98 and the Interaction Between CircNUP98 and Pre-mature miR-519a-3p

**Figure 2:**
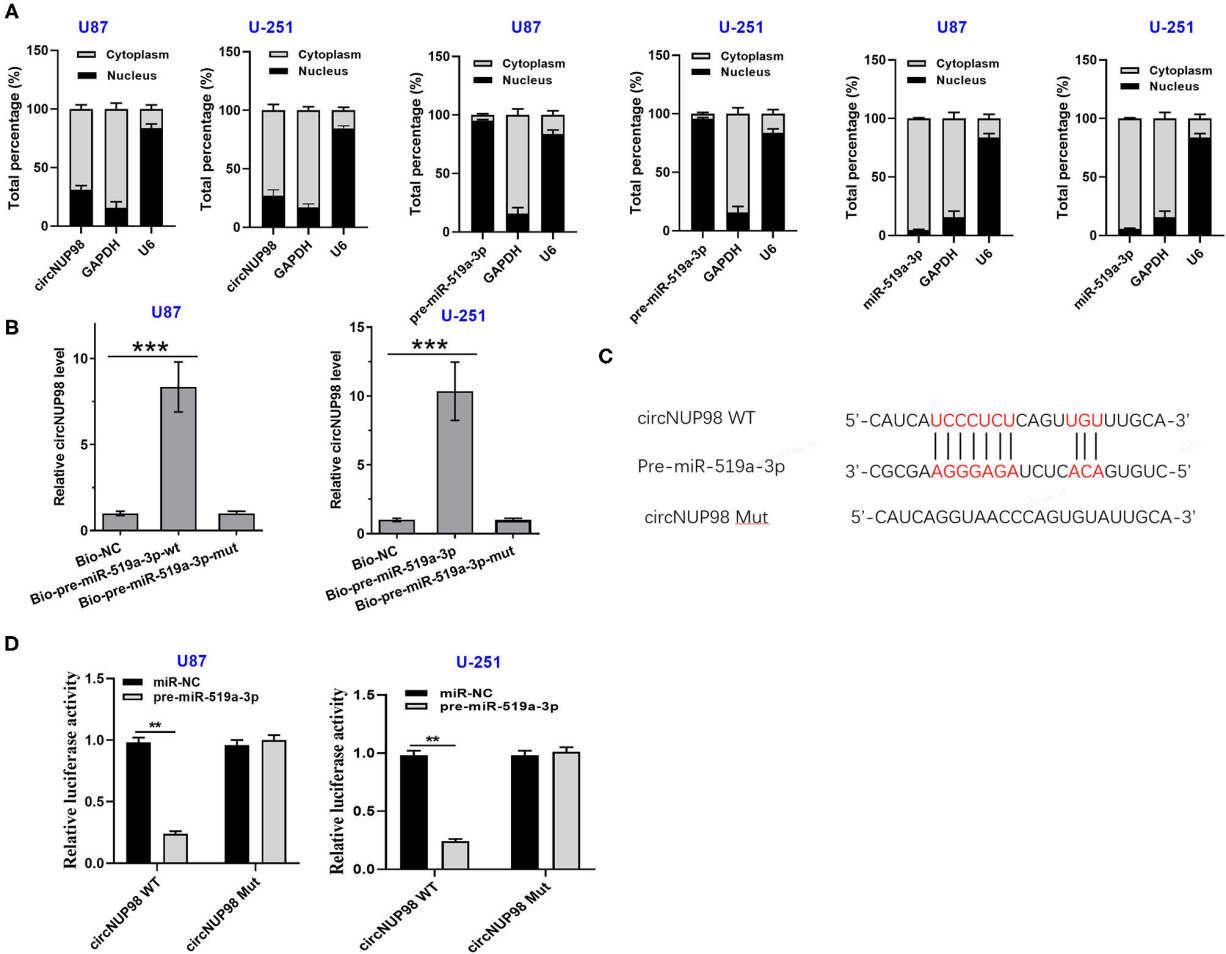
The subcellular location of circNUP98 and the interaction between circNUP98 and pre-mature miR-519a-3p. Nuclear fractionation assay was performed to analyze the expression of circNUP98 and pre-mature and mature miR-519a-3p in both the nucleus and cytoplasm from U87 and U-251 cells was analyzed **(A)**. The potential target between circNUP98 and pre-mature miR-519a was predicted by the StarBase version 2.0 Software (http://starbase.sysu.edu.cn/mirLncRNA.php). RNA pull-down assay was performed **(B)**. RNA pull-down assay **(C)** and dual-luciferase assay **(D)** performed to analyze the interaction between circNUP98 and pre-mature miR-519a-3p. *n* = 3; **p < 0.01 and ***p < 0.001. Bio-NC, biotinylated negative control; Bio-pre-miR-519a-3p, biotinylated pre-mature miR-519a-3p; Bio-pre-miR-519a-3p-Mut, biotinylated pre-mature miR-519a-3p mutant type; WT, wild type; Mut, mutant type.

Nuclear fractionation assay showed that circNUP98 localized into both the nucleus and cytoplasm and pre-mature miR-519a-3p mainly localized into the nucleus, while mature miR-519a-3p mainly localized into the cytoplasm (Figure 2A). In addition, U87 and U-251 cells were transfected with bio-pre-miR-519a-3p-WT or bio-miR-519a-3p-Mut or bio-NC. RNA pull-down assay showed that the enrichment of circNUP98 was higher in bio-pre-miR-519a-3p-WT group than that in bio-NC group, while there is no significant difference between bio-pre-miR-519a-3p-Mut and bio-NC groups (Figure 2B). Moreover, pre-miR-519a-3p was predicted to interact with circNUP98 in the StarBase version 2.0 software (Figure 2C). Dual-luciferase assay showed that miR-519a-3p overexpression obviously enhanced the luciferase activity in GB cells transfected with circNUP98 WT, while it had no effect on the luciferase activity in GB cells transfected with circNUP98 Mut (Figure 2D). These data indicating that circNUP98 interacts with pre-mature miR-519a-3p.

### Role of CircNUP98 in the Maturation of miR-519a-3p

**Figure 3:**
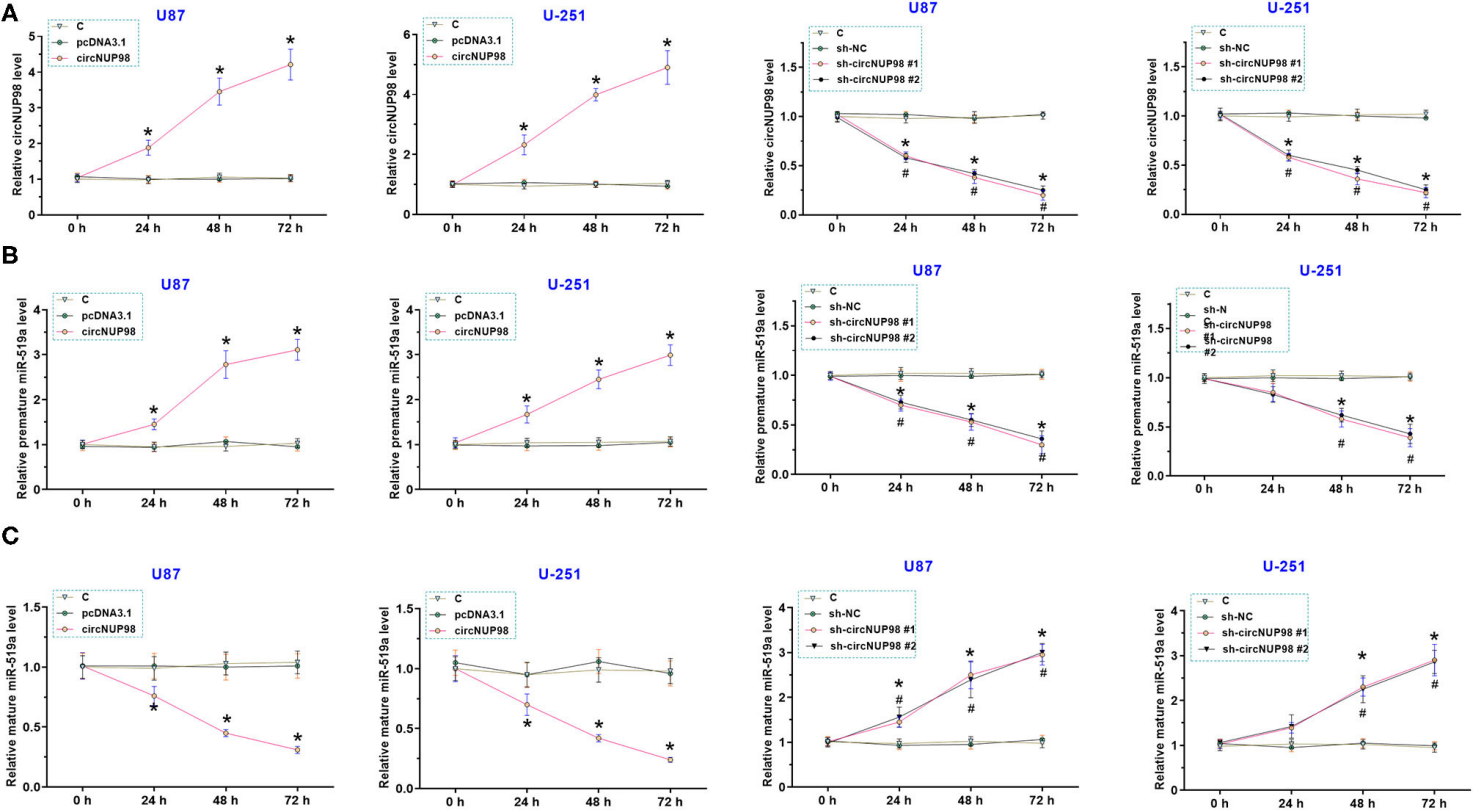
The role of circNUP98 in the maturation of miR-519a-3p. CircNUP98 was overexpressed and knocked down in U87 and U-251 cells by transfecting with circNUP98 overexpression vector and short hairpin RNA (shRNA), respectively. The expression levels of circNUP98 **(A)**, pre-mature miR-519a-3p **(B)**, and mature miR-519a-3p **(C)** at 24, 48, and 72 h after transfection by RT-qPCR assay. *n* = 3; *p < 0.05. C, control group.

To overexpress and knock down circNUP98, U87 and U-251 cells were transfected with circNUP98 overexpression vector and circNUP98 shRNA, respectively. We found that the circNUP98 level was significantly upregulated in the cells transfected with circNUP98 overexpression vector and it was obviously downregulated in the cells transfected with circNUP98 shRNA (Figure 3A, *p* < 0.05). In addition, we also detected the expression levels of pre-mature and mature miR-519a-3p. Results showed that circNUP98 overexpression obviously upregulated the level of pre-mature miR-519a-3p, while circNUP98 knockdown downregulated the level of pre-mature miR-519a-3p (Figure 3B, *p* < 0.05). On the contrary, mature miR-519a-3p level was downregulated by circNUP98 overexpression, while its level was upregulated by circNUP98 knockdown (Figure 3C, *p* < 0.05). These data indicating that circNUP98 directly interacts with pre-mature miR-519a-3p.

### Role of CircNUP98 and miR-519a-3p in the Proliferation of GBM Cells and Xenograft Mouse Model *in vivo*

**Figure 4:**
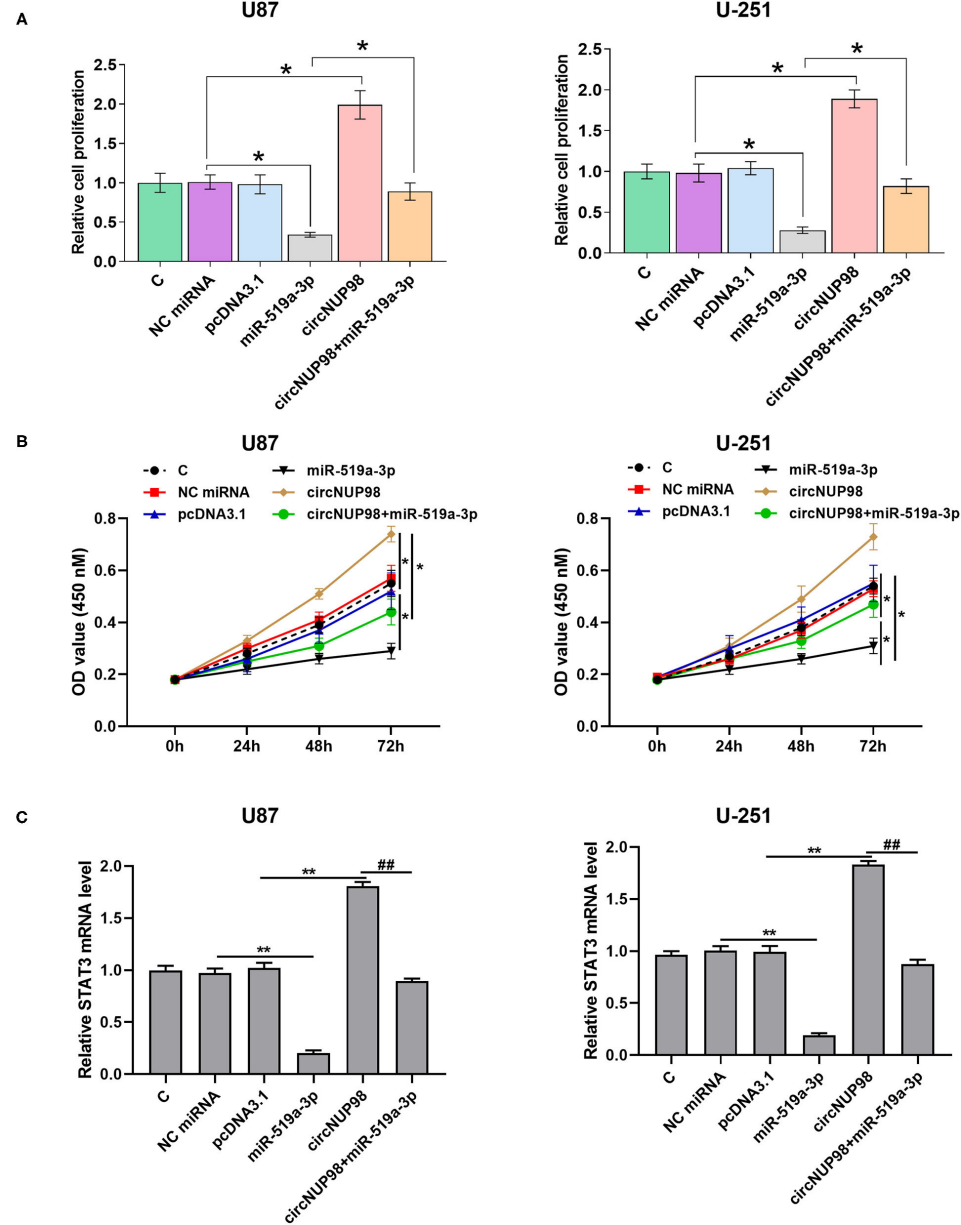
The role of circNUP98 and miR-519a-3p in the proliferation of GBM cells. GB cells were transfected with circNUP98 overexpression vector and miR-519a-3p mimics. BrdU assay was performed to detect the proliferation of GB cells **(A)**. CCK-8 assay was performed to detect the GB cell viability **(B)**. The STAT3 mRNA level was detected by RT-qPCR assay **(C)**. *n* = 3; *p < 0.05; **p < 0.01; ^##^*p* < 0.01. C, control group; NC miRNA, negative control of miRNA.

BrdU assay and CCK-8 assay were performed to explore the role of circNUP98 in the proliferation of GBM cells. It was observed that overexpression of circNUP98 increased cell proliferation and viability, while overexpression of miR-519a-3p decreased cell proliferation and viability (Figures 4A,B, *p* < 0.05). Moreover, overexpression of miR-519a-3 produced the effects of circNUP98 on cell proliferation and viability (Figures 4A,B, *p* < 0.05). In addition, the expression levels of STAT3, a direct target of miR-519a-3p, was detected by RT-qPCR. Results showed that overexpression of circNUP98 increased the level of STAT3 mRNA, while overexpression of miR-519a-3p reduced the level of STAT3 mRNA (Figure 4C, *p* < 0.05). Moreover, overexpression of miR-519a-3p attenuated the effect of circNUP98 overexpression on STAT3 expression (Figure 4C, *p* < 0.05).

**Figure 5:**
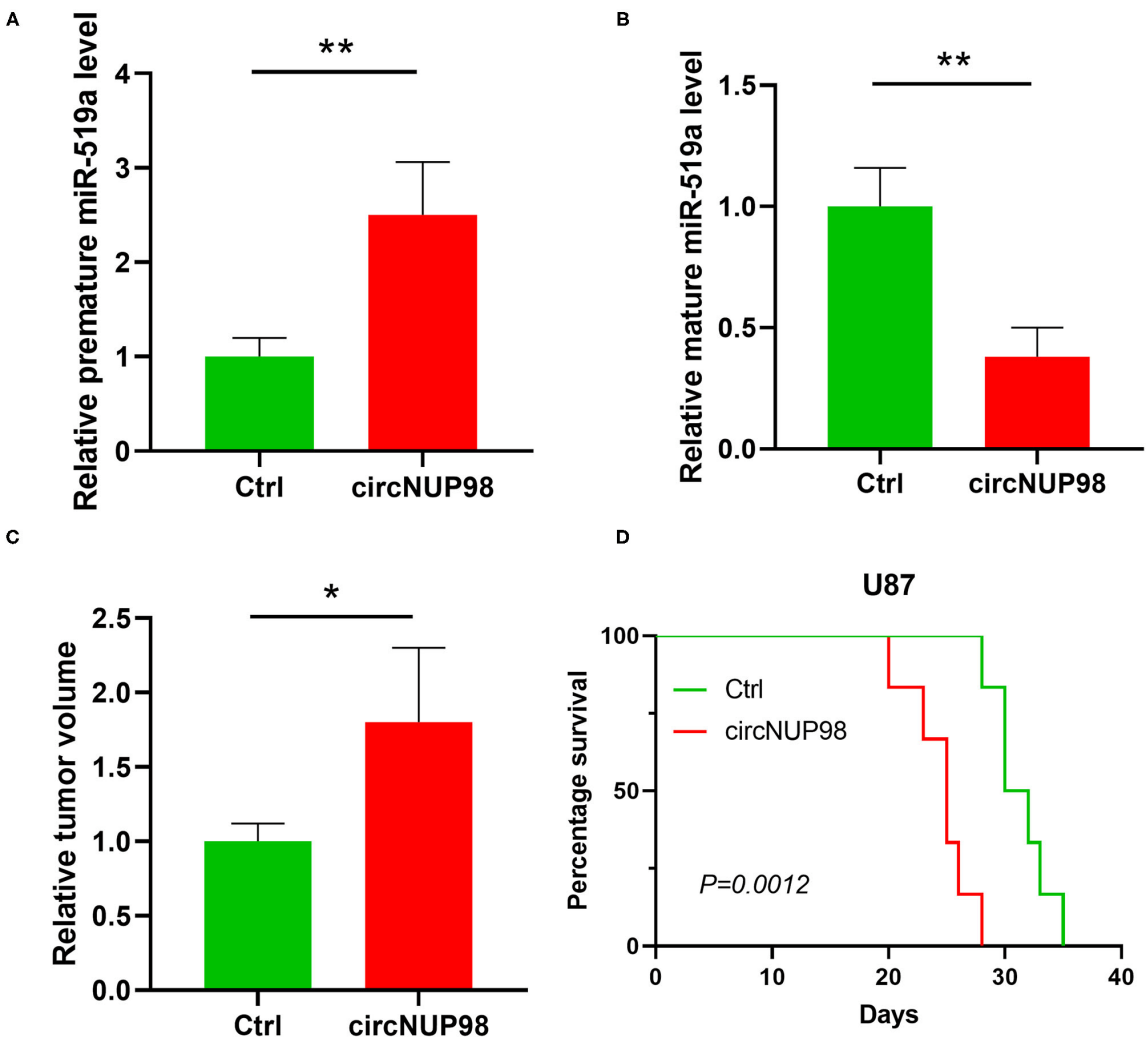
The role of circNUP98 and miR-519a-3p in GBM *in vivo*. The role of circNUP98 in pre-mature miR-519a-3p expression **(A)** and mature miR-519a-3p expression **(B)** was analyzed by RT-qPCR assay in GBM tissue (U87 and circNUP98 U87) from intracranial xenograft mice. Relative tumor volume **(C)** was measured and the survival of xenograft mice was analyzed by the Kaplan–Meier curve **(D)** to assess the role of circNUP98 *in vivo*. *p < 0.05, **p < 0.01. Ctrl, control.

To further confirm the role of circNUP98 in GB, mice were intracranially transplanted with control U87 cell line or U87 cell line with the overexpression of circNUP98. We observed that circNUP98 increased the expression levels of pre-mature miR-519a-3p in tumor tissues (Figure 5A), whereas decreased the expression levels of mature miR-519a-3p (Figure 5B). As shown in Figure 5C, overexpression of circNUP98 caused lager tumors compared with that in the control (*p* < 0.05), which resulted in significantly reduced mice survival (Figure 5D, *p* < 0.01). These results indicated the role of circNUP98 and miR-519a in tumorigenesis.

## Discussion

This study explored the involvement of circNUP98 and its interaction with miR-519a-3p in GBM. We found that circNUP98 was upregulated and miR-519a-3p maturation was significantly suppressed in GBM tissues. Moreover, circNUP98 could suppress the maturation of miR-519a-3p in GBM cells to increase cell proliferation in xenograft mouse model.

CircNUP98 is a recently characterized circRNA with known functions only in RCC (17). It was observed that STAT3 induces the upregulation of circNUP98, which, in turn, downregulates miR-567 to upregulate Peroxiredoxin III (PRDX3), thereby increasing cell proliferation and suppressing cell apoptosis (17). This study mainly explored the expression pattern and functionality of circNUP98 in GBM. We found that upregulation of circNUP98 resulted in increased proliferation rate of GBM cells. Our results also showed that overexpression of circNUP98 resulted in significantly reduced mice survival. Our data suggested that circNUP98 is an oncogenic circRNA in GBM. However, the potential of circNUP98 in the diagnosis and treatment of GBM remains to be further explored.

A recent study reported the downregulation of miR-519a-3p in GBM and its role in enhancing chemosensitivity and promoting cancer cell autophagy via targeting the STAT3/B cell lymphoma 2 (Bcl2) signaling (22). Consistently, our study confirmed the downregulation of mature miR-519a-3p in GBM. Interestingly, we also observed the upregulation of pre-mature miR-519a-3p. Therefore, the inhibited maturation of miR-519a-3p, but not the reduced transcription of miR-519a-3p, is involved in the pathogenesis of GBM.

Although the downstream targets of miR-519a-3p, such as STAT3/Bcl2, have been well-studied in cancer biology (22), its upstream regulators remain largely unknown. In this study, we showed that overexpression of circNUP98 could increase the expression levels of pre-mature miR-519a-3p and decrease the expression levels of mature miR-519a-3p in GBM cells. Therefore, circNUP98 could suppress the maturation of miR-519a-3p. Interestingly, we also observed the interaction between circNUP98 and pre-mature miR-519a-3p. However, circNUP98 and pre-mature miR-519a-3p could directly interact with each other or through other mediators. In addition, circNUP98 is localized to both the nucleus and cytoplasm of GBM cells. Pre-mature miRNAs are exclusively localized in nucleus and mature miRNAs are only found in cytoplasm (23). To form mature miRNAs, pre-mature miRNAs have to be transported out of the nucleus to cytoplasm. Therefore, we speculated that circNUP98 may sponge pre-mature miR-519a-3p in nucleus to suppress its transportation to cytoplasm, thereby reducing the maturation.

Previous studies have found that circRNAs can be translatable and circRNA-encoded functional proteins are involved in tumorigenesis (24). A recent study has reported that a circular E-cadherin (circ-E-Cad) RNA can promote GB tumorigenicity via activating EGFR–STAT3 pathway by encoding an oncogenic E-cadherin variant (25). CircNUP98, as a circRNA, might be also function in GBM by encoding a functional protein, which deserves to be further explored in our future study.

However, this study has some limitations. Firstly, our sample size is not big enough. Our future study will collect more samples to support our findings. In addition, this study only investigated the effect of circNUP98 on GB cell proliferation. CircNUP98 may also function in GB by regulating other biological processes, which deserved to be further explored.

## Conclusion

CircNUP98 was highly upregulated in GBM tissues and it may sponge pre-mature miR-519a-3p in nucleus to suppress it maturation, thereby increasing cell proliferation.

## Data Availability Statement

The original contributions presented in the study are included in the article/supplementary material, further inquiries can be directed to the corresponding author/s.

## Ethics Statement

The studies involving human participants were reviewed and approved by Taizhou People's Hospital. The patients/participants provided their written informed consent to participate in this study.

## Author Contributions

XW and JJ contributed to the guarantor of integrity of the entire study and study concepts. JL contributed to the study design, study perform, and original manuscript writing. GL, LJ, and XL contributed to the definition of intellectual content, literature research, experimental study, and data acquisition. All authors contributed to the article and approved the submitted version.

## Conflict of Interest

The authors declare that the research was conducted in the absence of any commercial or financial relationships that could be construed as a potential conflict of interest.

## Publisher's Note

All claims expressed in this article are solely those of the authors and do not necessarily represent those of their affiliated organizations, or those of the publisher, the editors and the reviewers. Any product that may be evaluated in this article, or claim that may be made by its manufacturer, is not guaranteed or endorsed by the publisher.
